# Differential diagnosis of psychosis and dissociative disorder: a case report

**DOI:** 10.1192/j.eurpsy.2023.1578

**Published:** 2023-07-19

**Authors:** E. Arroyo Sánchez, P. Setién Preciados

**Affiliations:** Psiquiatría, Hospital Universitario Príncipe de Asturias, Madrid, Spain

## Abstract

**Introduction:**

Psychosis and dissociative disorders are both described in the DSM-5 as different diagnostic categories. However, a high comorbidity of these diagnoses has been observed in different studies, perhaps due to the overlapping of symptoms between them.

**Objectives:**

A systematic review about overlapping symptoms in psychotic spectrum and dissociative disorders

**Methods:**

Presentation of the case of a patient and review of the existing literature on the differential diagnosis between dissociative disorder and other psychotic spectrum disorders.

**Results:**

Both similarities and differences have been found between both diagnoses. Patients with dissociative disorder experienced more dissociative and positive symptoms while those on the psychotic spectrum experienced more negative symptoms. The literature reflects that the two entities overlap on many of their diagnostic symptoms. On some occasions, more dissociation has been detected in patients diagnosed with the psychotic spectrum than those with a diagnosis of dissociative disorder.

**Conclusions:**

Despite the fact of being different diagnostic entities, the literature does not reflect clear boundaries between psychosis spectrum symptoms and dissociative disorder.

**Disclosure of Interest:**

None Declared

